# Trichloroethylene and Its Oxidative Metabolites Enhance the Activated State and Th1 Cytokine Gene Expression inJurkat Cells

**DOI:** 10.3390/ijerph120910575

**Published:** 2015-08-28

**Authors:** Yao Pan, Xuetao Wei, Weidong Hao

**Affiliations:** 1 Department of Toxicology, School of Public Health, Peking University, Beijing 100191, China; E-Mails: yaopan408@gmail.com (Y.P.); weixt@263.net (X.W.); 2 Beijing Key Laboratory of Toxicological Research and Risk Assessment for Food Safety, Beijing 100191, China

**Keywords:** trichloroethylene, trichloroacetic acid, dichloroacetic acid, IL-2, Jurkat cells

## Abstract

Trichloroethylene (TCE) is an occupational and ubiquitous environmental contaminant, and TCE exposure will increase the risk of autoimmune diseases and allergic diseases. T cells play an important role in the pathogenesis of TCE-related immune disorders, but the effect of TCE and its oxidative metabolites, trichloroacetic acid (TCA) and dichloroacetic acid (DCA), on the activation of human T cells is still unknown. In this study, Jurkat cells were pre-treated with TCE, TCA and DCA overnight and then stimulated with phorbol 12-myristate 13-acetate and ionomycin for another 4, 8 and 24 hours. IL-2 secretion was detected by ELISA; the expressions of CD25 and CD69 were tested by flow cytometry; and IFN-γ and IL-2 mRNA expression levels were investigated by real-time PCR. The results showed that TCE and its oxidative metabolites, TCA and DCA, significantly enhanced IL-2 releasing and the expression of T cell activation markers, CD25 and CD69. Consistent with this result, these compounds markedly up-regulated the expression levels of IFN-γ and IL-2 mRNA. Collectively, these findings suggest that TCE and its metabolites, TCA and DCA, might enhance the activation of T cells and disrupt various activities of peripheral T cells.

## 1. Introduction

Trichloroethylene (TCE) is a chlorinated solvent once widely used as a metal degreaser, chemical intermediate and extractant, and now, it has become an occupational and ubiquitous environmental contaminant. Human and laboratory animal studies of TCE and immune-related effects provide strong evidence that TCE is a potent immunomodulator that accelerates the development of allergic diseases [1,2] and autoimmune diseases [3,4,5]. TCE-induced immunological diseases have been associated with both occupational exposure and environmental exposure, primarily through groundwater contamination [6]. Many case reports have described a severe hypersensitivity skin disorder, which is often accompanied by hepatitis, and its prevalence is as high as 13% of workers in the same working location [7,8]. Although the pathogenesis of this hypersensitivity skin disorder is still unclear, most dermatologists and physicians in occupational medicine categorize it as a kind of Tcell-mediated delayed-type hypersensitivity reaction [9].

Most of the toxicological effects of TCE require its metabolism [10]. The majority of TCE is metabolized through a cytochrome P450 (CYP) pathway involving the enzyme CYP2E1 into oxidative byproducts, including trichloroacetaldehyde (TCAH), trichloroaceticacid (TCA) and dichloroaceticacid (DCA) [11]. As the metabolic intermediate of TCE, there were numerous *in vivo* and *in vitro* studies on TCAH [12,13,14]. However, how the downstream metabolites, TCA and DCA, act in the pathological process caused by TCE is still unknown.

Due to the important role of T cells in the immune response, in this study, we analyzed the impact of TCE and its oxidative metabolites, TCA and DCA, on human T lymphocytes, using human T lymphoma cell line Jurkat E6-1. The Jurkat cell line is one of the most widely-used cellular T cell models, which shares numerous features with naive T cells. Like normal human T cells, induction of IL-2 secretion can be triggered upon receiving two signals: one through the T cell receptor (TCR) and the second through the costimulatory molecule CD28 [3,15,16]. This stimulation can also be mimicked by mitogen phorbol 12-myristate 13-acetate (PMA) and ionomycin (Ion), which is another similarity between naive and Jurkat T cells [17,18].Using different stimulation time points offers a way to analyze the toxic effects of TCE and its oxidative metabolites, TCA and DCA, on the dynamic process of activation. As the activation of T cells by antigens is a crucial process in the immune response, the effect of TCE and its oxidative metabolites on this process is of special interest.

The objective of this study was to identify the effect of TCE and its oxidative metabolites, TCA and DCA, on the early events of T cell activation in a human T cell model. Furthermore, the expression of IFN-γ and IL-2 mRNA, which are the signature cytokines of T helper 1 (Th1) cells, was detected to investigate the impact of TCE and its metabolites on the potential to induce T cell differentiation.

## 2. Materials and Methods

### 2.1. Chemicals, Antibodies and Supplies

Trichloroethylene, trichloroacetic acid, dichloroacetic acid, phorbol 12-myristate 13-acetate, ionomycin and dimethyl sulfoxide (DMSO) were obtained from Sigma (St. Louis, MO, USA). RPMI-1640 medium, penicillin/streptomycin, hydroxyethyl piperazineethanesulfonic acid (HEPES), L-glutamine and phosphate-buffered saline (PBS) (without Ca^2+^ and Mg^+^) were obtained from Invitrogen (GibcoBrand; Carlsbad, CA, USA). Fetal bovine serum (FBS) was purchased from Sigma (St. Louis, MO, USA). The human IL-2 Ready-SET-Go! ELISA (2nd Generation)-Kit was obtained from eBioscience (San Diego, CA, USA). Anti-human CD25 PE and CD69 APC antibodies were purchased from BD Pharmingen (San Diego, CA, USA). The CellTiterGlo^®^ luminescent cell viability assay was purchased from Promega (Madison, WI, USA). The RNeasy mini kit was purchased from Qiagen (Valencia, CA, USA). The qScript One-Step Fast qRT-PCR kit was obtained from Quanta BioSciences (Gaithersburg, MD, USA). Flat-bottomed 96-well plates, 24-well plates and other disposables were obtained from Fisher Scientific (Atlanta, GA, USA).

### 2.2. Cell Culture

Jurkat cells (clone E6-1, TIB-152) were received from the American Type Culture Collection (ATCC, Manassas, Virginia). Cells were cultured in 75-cm^2^ tissue culture flasks in RPMI-1640 medium supplemented with10% FBS, 500μg/mL penicillin/streptomycin, 5 mM HEPES and 2mML-glutamine and incubated under a humidified atmosphere of 5% CO_2_/95% air at 37 °C. Growth medium was changed every 2 days. For the experiments, cells were seeded in complete medium and treated with each compound or vehicle (final 0.5%, v/v).

### 2.3. Assessment of Cell Viability

Jurkat cells were plated in triplicate per dose on 96-well plates at 5 × 10^4^ cells per well and stimulated with the combination of 50ng/mL PMA and 1mM Ion. For TCE, TCA and DCA treatments, compound stock solutions were prepared in DMSO and diluted in cell culture media to the indicated final concentrations. The final DMSO concentration was 0.5% in vehicle control wells. Cells were incubated for 4 h and 24h at 37 °C. At 4h and 24 h post-compound exposure, cell viability was measured using the CellTiterGlo luminescent cell viability assay following the manufacturer’s instruction. The bioluminescence was measured by a Safire2 microplate reader (Tecan, Switzerland). Data represent the mean measurements from triplicate treatments.

### 2.4. Measurement of IL-2 Production

Jurkat cells were seeded on 24-well plates at 3 × 10^5^ cells per well and treated with the indicated final concentrations of TCE, TCA and DCA for 16 h. Then, cells were stimulated with 50 ng/mL PMA and 1mM Ion and incubated at 37 °C for another 4 h, 8 h and 24 h. The cell supernatants were collected and measured for IL-2 concentration using the human IL-2 ELISA Ready-SET-Go (2nd generation)-Kit following the manufacturer’s advice.

### 2.5. Detection of Activation Markers

Jurkat cells were seeded on 24-well plates at 3 × 10^5^ cells per well and treated with the indicated final concentrations of TCE, TCA and DCA for 16h. Then, cells were stimulated with 50 ng/mL PMA and 1mM Ion and incubated at 37 °C for another 2 h, 4 h and 24 h. Jurkat cells were harvested and washed with ice-cold PBS. Cells were incubated with 20μL anti-human CD25 PE and CD69 APC antibodies in 100 μL staining buffer (2%FBS in PBS) at 4 °C for 30 min in the dark. Excessive unreacted antibodies were removed by washing with staining buffer. Cells were resuspended in 300 μL staining buffer and analyzed with flow cytometry (Accuri C6, BD Biosciences).

### 2.6. Real-Time PCR Analysis

After 4 h post-stimulation, Jurkat cells (3 × 10^5^) were harvested and washed with ice-cold PBS. Cells were lysed in RLT buffer, and total RNA was isolated using the RNeasy mini kit. The primers and probes used in this study were human IFN-γ (Assay ID: Hs00989291_m1), IL-2 (Assay ID: Hs00174114_m1) and GAPDH (Assay ID: Hs02758991_g1).Taqman^®^ Gene Expression Assays were purchased from Applied Biosystems (Carlsbad, CA, USA). The quantification of the target mRNA was performed on a LightCycler 480 Real-Time PCR System (Roche Applied Science, Indianapolis, IN). The data were analyzed using the comparative Ct (threshold cycle) method, and GAPDH was the reference gene.

### 2.7. Statistical Analysis

All experiments were repeated at least three times, with representative results shown. Statistical analysis was performed using GraphPad Prism software (GraphPad, La Jolla, CA, USA). Data are expressed as the mean ± standard deviation (SD). Statistical significance was determined using a one-way ANOVA (*p* < 0.05) followed by Dunnett’s comparison to compare treatment groups to controls.

## 3. Results

### 3.1. Effect of TCE, TCA and DCA on the Cell Viability of Jurkat Cells

**Figure 1:**
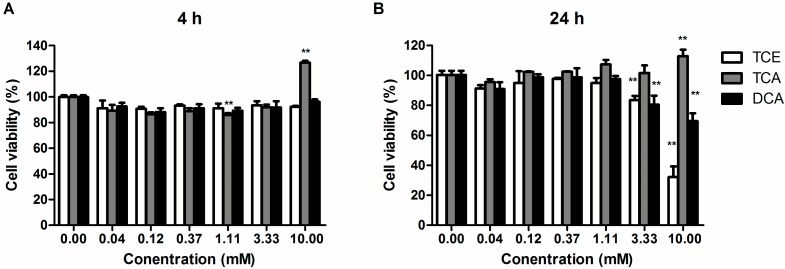
Effect of trichloroethylene (TCE), trichloroacetic acid (TCA) and dichloroacetic acid (DCA) on the cell viability of Jurkat cells. Cells were treated with TCE, TCA and DCA combined with the stimulation of phorbol 12-myristate 13-acetate (PMA) (50 ng/mL) and ionomycin (Ion) (1 mM) for 4 h (**A**) and 24 h (**B**) at 37 °C. Cell viability was then determined by the CellTiterGlo^®^ luminescent cell viability assay, and the results are shown as the mean ± SD of three independent experiments. ^**^ *p* < 0.01 compared to the DMSO vehicle control group.

To determine the effect of TCE, TCA and DCA on cell viability, Jurkat cells were stimulated with PMA/Ion in the presence of TCE, TCA, DCA or vehicle (DMSO). DMSO exposure had no effect on the ability of Jurkat cells to respond to PMA/Ion when compared to cells stimulated in the absence of DMSO (data not shown). Figure 1A shows the result of the CellTiterGlo^®^ luminescent cell viability assay after 4 h of compound treatment. With PMA/Ion stimulation, concentrations of TCE and DCA ranging from 0.04 to 10.00 mM did not exhibit any cytotoxic effect compared to the DMSO group, as their cell viability values were all above 90%. However, the cell viability of TCA-treated Jurkat cells decreased at first to the lowest value of 85.82% at 1.11 mM and then increased to 125.59% at 10.00 mM. Figure 1B shows the cell viability result after 24h of compound treatment. A dose-effect increase in the cytotoxicity of TCE or DCA was shown in the result, and treatment of Jurkat cells with 3.33 mM TCE or DCA caused significant cell death (*p* < 0.01), with a relative cell viability around 80%. TCE cytotoxicity to Jurkat cells was comparable to DCA. In the present study, we chose 3.0 mM TCE or DCA as the maximum treatment concentration to do further study. On the contrary, the cell viability of TCA-treated Jurkat cells went up with the rising dose, and at 10.00 mM, the cell viability markedly rose to about 112.89%. However, as a strong acid, the concentrations of TCA lower than 1.0 mM kept the pH value of the cell culture medium within 7.2 and 7.4 (data not shown).Therefore, we chose 1.0 mM TCA as the maximum treatment concentration to do further study.

### 3.2. TCE, TCA and DCA Enhanced the Production of IL-2 in Jurkat Cells

**Figure 2:**
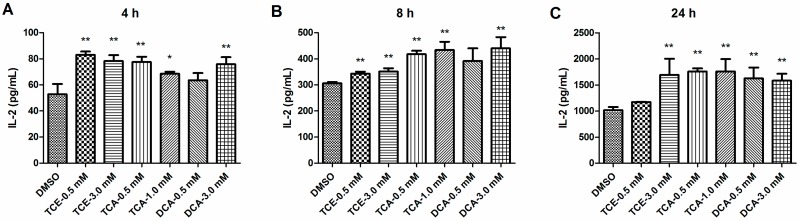
Effect of TCE, TCA and DCA on the production of IL-2 in PMA-/Ion-stimulated Jurkat cells. Cells were pretreated with TCE (0.5 and 3.0 mM), TCA (0.5 and 1.0 mM) and DCA(0.5 and 3.0 mM) for 16 h and then stimulated with PMA(50 ng/mL) and Ion (1 mM) for 4 h, 8 h and 24 h. The amounts of IL-2 in the cell supernatants after PMA/Ion stimulation for 4 h (**A**), 8 h (**B**) and 24 h (**C**) were measured by ELISA. The bars represent the mean ± SD of three independent experiments. ^*^ *p* < 0.05, ^**^ *p* < 0.01 compared to the DMSO vehicle control group.

IL-2 is mainly produced by activated Jurkat T cells, and the quantity of synthesized IL-2 is an important measure of the degree of cell activation. We subsequently examined whether TCE, TCA or DCA could raise the production of IL-2 at the non-cytotoxic concentrations. Jurkat cells were treated with TCE, TCA and DCA for 16h and then stimulated with PMA/Ion for 4 h, 8 h and 24 h, and IL-2 production was determined by the enzyme immunoassay. As shown in Figure 2A to 2C, pretreatment of Jurkat cells with 3.0 mM TCE, 0.5 and1.0 mM TCA and 3.0 mM DCA for 16h significantly enhanced PMA-/Ion-induced production of IL-2 at all-time points. Pretreated with 0.5mM TCE and then stimulated by PMA/Ion for 4h and 8h, Jurkat cells released markedly more IL-2, but this trend vanished after extending the stimulation time to 24h. In contrast, pretreatment of 0.5 mM DCA had no effect on IL-2 production in Jurkat cells activated with PMA/Ion for 4h and 8h, whereas a significant increasing effect occurred after 24 h activation. These data suggested that TCE, TCA and DCA modulated cytokine production in human T cells.

### 3.3. Influence of TCE, TCA and DCA on JurkatCells Activation Status

**Figure 3:**
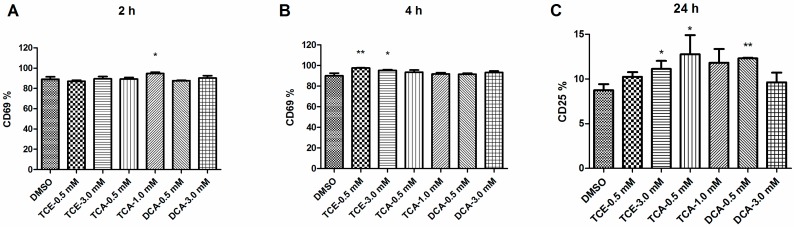
Effect of TCE, TCA and DCA on CD25 and CD69 expression levels in Jurkat cells. Cells were pretreated with TCE (0.5 and 3.0 mM), TCA (0.5 and 1.0 mM) and DCA (0.5 and 3.0 mM) for 16h and then stimulated with PMA (50ng/mL) and Ion (1mM) for 2 h, 4 h and 24 h. Jurkat cells were isolated and labeled with anti-human CD25 PE and CD69 APC antibodies. CD25 and CD69 expressions were analyzed by flow cytometry. Graphical representation of CD69-positive cells by flow cytometry after PMA/Ion stimulation for 2 h (**A**) and 4 h (**B**) and graphical representation of CD25-positive cells by flow cytometry after PMA/Ion stimulation for 24h (**C**). The bars represent the mean ± SD of three independent experiments. ^*^ *p* < 0.05, ^**^ *p* < 0.01 compared to the DMSO vehicle control group.

Because TCE, TCA and DCA markedly increased IL-2 production by activating Jurkat cells (Figure 2), we next investigated the influence of TCE, TCA and DCA on other early events of T cell activation. CD69, a lectin-type receptor, is a marker of very early T cell activation [19]. Poststimulation by PMA/Ion for 2h, only the 1.0 mM TCA group showed a statistically relevant increase in CD69 expression (Figure 3A). Prolonging the stimulation time to 4 h, the percentages of CD69-positive cells in all groups were over 90%, and only the TCE (0.5 and 3.0 mM) treatment group slightly enhanced CD69 expression (Figure 3B). After PMA/Ion stimulation for 24 h, the CD69-positive cell rates in all groups reached 100%, so we compared the CD25 expression level instead. In addition to CD69, the high affinity α-chain of the IL-2 receptor (CD25) is also an early activation marker in T cells [20]. Pretreatment of Jurkat cells with 3.0 mM TCE, 0.5mM TCA and 0.5 mM DCA for 16 h could significantly increase CD25 expression (Figure 3C).

### 3.4. TCE, TCA and DCA Upregulated Th1 Cytokine Gene Expression in Jurkat Cells

**Figure 4:**
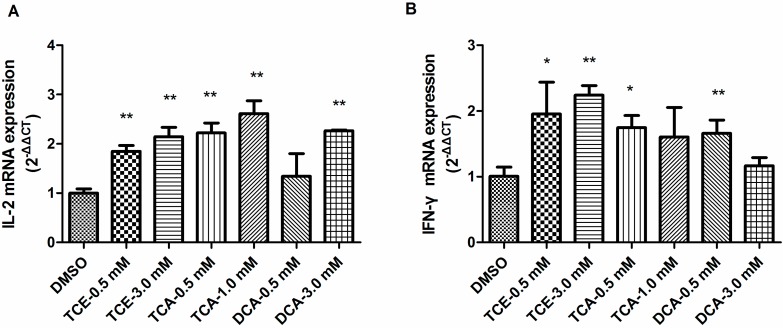
Effect of TCE, TCA and DCA on Th1 cytokine gene expressions in Jurkat cells. Cells were pretreated with TCE (0.5 and 3.0 mM), TCA (0.5 and 1.0 mM) and DCA(0.5 and 3.0 mM) for 16h and then stimulated with PMA(50ng/mL) and Ion (1 mM) for 4 h. The IL-2 (**A**) and IFN-γ (**B**) mRNA expression levels were analyzed by real-time PCR. The bars represent the mean ± SD of three independent experiments. ^*^ *p* < 0.05, ^**^ *p* < 0.01 compared to the DMSO vehicle control group.

As TCE, TCA and DCA could promote IL-2 releasein Jurkat cells (Figure 2), we sought to study whether the signature cytokine gene expressions of Th1 cells, IFN-γ and IL-2, were affected by those compounds in PMA/Ion-stimulated Jurkat cells. The real-time PCR analysis indicated that two treatment groups of TCE and TCA and 3.0 mM DCA could dramatically increase IL-2 mRNA expression (Figure 4A). As shown in Figure 4B, except 1.0 mM TCA and 3.0 mM DCA, other compound treatment groups significantly enhanced the IFN-γ mRNA expression level.

## 4. Discussion

Trichloroethylene hypersensitivity syndrome (THS) is becoming a serious occupational disease in China among workers exposed to TCE, and there were 394 cases of THS that have been diagnosed in Guangdong Province in Southern China from 1988 to 2010 [21]. The guinea pig maximization test (GPMT) showed that TCE itself is a strong allergen and that TCA is a moderate one [1]. In addition, patch tests performed in THS patients also had positive results using TCE and TCA as allergens [22,23,24,25], and these studies support that the T cell-mediated hypersensitivity reaction is the main immune reaction of THS. The first step leading to this reaction is the activation of antigen-specific inflammatory helper T (Th1) cells. Therefore, we hypothesized that not only TCE, but also its metabolites are responsible for the activation of T cells in the immune reaction of THS. In this study, we chose the Jurkat cell line as model cell to explore the effect of TCE and its two oxidative metabolites, TCA and DCA.

Activation of T cells plays an important role in steering the immune responses to perform immune functions. One of the most important markers of Jurkat cells activation is the increasing IL-2 secretion, and this IL-2 is not taken by Jurkat cells to maintain its survival [26]. Another result of T cell activation is that the expressions of the canonical T cell activation markers, CD25 and CD69, are upregulated [27]. These early events of Tcell activation are vital in initiating an effective immune response. In the present study, we found that 3.0mM TCE and DCA and 0.5mM and 1.0mM TCA increased the IL-2 secretion of Jurkat cells at all-time point, and the corresponding IL-2 mRNA expression was also upregulated in these treatment groups. Moreover, the effect of 0.5mM TCE on inducing IL-2 production in Jurkat cells occurred at 4h and 8h, but 0.5mM DCA had this effect at 24 h. However, the impact of TCE, TCA and DCA on the expression of cell surface activation markers was diverse at different time points. TCE and TCA cause high expression of both CD69 and CD25, while DCA only upregulated CD25 expression at 24h. We used the mRNA level of IFN-g to exhibit the potential of Jurkat cells differentiating towards the Th1 phenotype, as Th1 cells are characterized by the cytokine IFN-g secretion. The upregulation of IFN-g mRNA expression was seen in all compound treatment groups compared to the vehicle control groups, but 1.0mM TCA and 3.0mM DCA did not show statistically significant increases. Taken together, these results indicated that TCE and its oxidative metabolites, TCA and DCA, all played some role in enhancing the activation state in Jurkat cells by inducing IL-2 expression at the protein and mRNA level, up-modulating the expression of T cell activation markers and in skewing Jurkat cells’ differentiation towards the Th1 phenotype through upregulating the mRNA expression level of IFN-γ. Nevertheless, TCE had an impact on all of the parameters detected in this study at almost all of the time point, while the effect of TCA was on increasing IL-2 secretion, and DCA seemed to affect the activation of Jurkat cells at a high dose and at later time points. In the human body, only part of TCE can be metabolized to TCA, and little DCA can enter into blood. On account of the concentrations of these three compounds that we chose were the same, the current study did not reflect the true exposure level *in vivo*. Further studies need to be done to discriminate the priority of TCE, TCA and DCA on activating Jurkat cells using different concentrations of each compound that are comparable to the human exposure levels.

In a series of studies of autoimmune-prone MRL +/+ mice exposed to TCE [28,29,30,31], TCA [32] and DCA [33], an acceleration of the autoimmune response and functional and phenotypic signs of T cell activation were indicated. Occupational TCE-exposed workers had higher IL-2 and IFN-γ levels, but lower IL-4 levels in serum [34], and high dose exposure to TCE from indoor air (defined as >75th percentile of the distribution) caused four-week-old infants to have an increase in the population of IFN-γ-secreting T cells and a decrease in IL-4-secreting T cells [35]. In these studies, it is still unknown what is the contributor of these TCE-induced human health hazards, the metabolites or TCE itself. *In vivo* studies are weak in defining the specific factors that cause the toxicity, whereas a cell line-based experimental system helps to provide insight into distinct cellular mechanism. Therefore, we set up a human T cell line model to figure out whether its TCE, the parent compound, caused the immunotoxicity or if this is owing to the oxidative metabolites. In this study, it was shown that both the parent compound and its two oxidative metabolites contributed to the toxicity effect. However, our findings in the current study mainly focused on the short-term TCE, TCA and DCA treatment of Jurkat cells, which was unable to reveal the effects under chronic TCE exposure. The concentrations (0.5 and 3.0 mM) we used to treat Jurkat cells in the study were relatively higher than the occupational exposure dose of TCE (*ca.* 22 ppm) [36,37]. Further efforts toward detailed relationships between the low-dose TCE exposure and its immunotoxicity should be explored, but this study offers some thoughtto the mechanism of how TCE induces toxicity in T cells. Measurement of TCA in urine is recommended as an indicator for monitoring the exposure to TCE among workers, and the amount of TCA in urine might, to a certain extent, reflect the exposure level *in vivo*. According to the report of 50 patients with THS in Guangdong Province, China, the range of urine TCA in 45 of them was 3.5 to 158.2 mg/L, which is equal to 0.02 to 0.97 mM [38]. The half-lives for TCA have ranged from 50 to over 100 h, depending on the compound administered [39,40]. Considering both of these aspects, we should pay attention to the effect of TCA on the immune system. Only a trace amount of DCA was detected in the blood of a few volunteers exposed to TCE [40] or chloral hydrate [41]. Therefore, the effect of DCA on activating T cells is debatable, as it took place at aconcentration far higher than the real exposure level.

There is a lack of published research on the immunotoxicity of the TCE metabolites, TCA and DCA, in human lymphocyte models. However, the present study is the first one exploring the effects of TCE and its metabolites on the early events of Jurkat cell activation and Th1 cytokine gene expressions. The observations above elucidate the role of TCE, TCA and DCA on enhancing the activation of T cells, which may provide some clues to interfere with TCE-related immune diseases.

## 5. Conclusions

The present study indicates that TCE and its metabolites, TCA and DCA, might enhance the activation of Jurkat cells and up-regulate its Th1 cytokine gene expression.
